# Does the time interval from neoadjuvant camrelizumab combined with chemotherapy to surgery affect outcomes for locally advanced esophageal squamous cell carcinoma?

**DOI:** 10.1007/s00432-024-05696-4

**Published:** 2024-03-27

**Authors:** Jiacong Liu, Linhai Zhu, Xuhua Huang, Zhongjie Lu, Yanye Wang, Yuhong Yang, Jiayue Ye, Chen Gu, Wang Lv, Chong Zhang, Jian Hu

**Affiliations:** 1 Department of Thoracic Surgery, The First Affiliated Hospital, School of Medicine, Zhejiang University, No. 79 Qingchun Road, Hangzhou, 310003 China; 2 Key Laboratory of Clinical Evaluation Technology for Medical Device of Zhejiang Province, Hangzhou, 310003 China

**Keywords:** Esophageal squamous cell carcinoma (ESCC), Locally advanced, Neoadjuvant immunochemotherapy, Survival, Interval time to surgery

## Abstract

**Background:**

There is currently no consensus on the optimal interval time between neoadjuvant therapy and surgery, and whether prolonged time interval from neoadjuvant therapy to surgery results in bad outcomes for locally advanced esophageal squamous cell carcinoma (ESCC). In this study, we aim to evaluate outcomes of time intervals ≤ 8 weeks and > 8 weeks in locally advanced ESCC.

**Methods:**

This retrospective study consecutively included ESCC patients who received esophagectomy after neoadjuvant camrelizumab combined with chemotherapy at the Department of Thoracic Surgery, the First Affiliated Hospital, Zhejiang University School of Medicine. The primary endpoints were disease-free survival (DFS) and overall survival (OS), while the secondary endpoints were pathological response, surgical outcomes, and postoperative complications.

**Results:**

From 2019 to 2021, a total of 80 patients were included in our study and were divided into two groups according to the time interval from neoadjuvant immunochemotherapy to surgery: ≤ 8 weeks group (*n* = 44) and > 8 weeks group (*n* = 36). The rate of MPR in the ≤ 8 weeks group was 25.0% and 27.8% in the > 8 weeks group (*P* = 0.779). The rate of pCR in the ≤ 8 weeks group was 11.4%, with 16.7% in the > 8 weeks group (*P* = 0.493). The incidence of postoperative complications in the ≤ 8 weeks group was 27.3% and 19.4% in the > 8 weeks group (*P* = 0.413). The median DFS in the two groups had not yet reached (hazard ratio [HR], 3.153; 95% confidence interval [CI] 1.383 to 6.851; *P* = 0.004). The median OS of ≤ 8 weeks group was not achieved (HR, 3.703; 95% CI 1.584 to 8.657; *P* = 0.0012), with the > 8 weeks group 31.6 months (95% CI 21.1 to 42.1). In multivariable analysis, inferior DFS and OS were observed in patients with interval time > 8 weeks (HR, 2.992; 95% CI 1.306 to 6.851; and HR, 3.478; 95% CI 1.481 to 8.170, respectively).

**Conclusions:**

Locally advanced ESCC patients with time interval from neoadjuvant camrelizumab combined with chemotherapy to surgery > 8 weeks were associated with worse long-term survival.

## Introduction

Esophageal cancer (EC) is the seventh most prevalent tumor and the sixth most common cause of cancer-related death worldwide (Siegel et al. 2022). Among its two histological subtypes, esophageal squamous cell carcinoma (ESCC) is more common in Asia, accounting for approximately 90% of EC cases in China. (Arnold et al. 2015; Zhang 2013). And most patients are diagnosed with locally advanced EC (Yang et al. 2023). Nowadays, the standard treatment for patients with locally advanced EC is neoadjuvant therapy [chemotherapy (Ando et al. 2012), chemoradiotherapy (Hagen et al. 2012; Yang et al. 2018), immunochemotherapy (Yan et al. 2022; Liu et al. 2022), chemoradiotherapy plus immunotherapy (Li et al. 2021)] followed by surgical resection. Neoadjuvant treatment can reduce the tumor size, lower the tumor stage, and subsequent surgical resection can remove the tumor more thoroughly and result in better outcomes.

Although neoadjuvant therapy followed by surgery has been recommended as the standard treatment for locally advanced EC, there still has been no consensus on the optimal interval time between neoadjuvant therapy and surgery. Surgical procedure was usually suggested after an interval of 4 to 8 weeks after completion of neoadjuvant treatment in current clinical studies (Hagen et al. 2012; Yang et al. 2018; Mukherjee et al. 2017; Haisley et al. 2016). However, surgical resection may sometimes be performed beyond this time frame owing to adverse events of neoadjuvant therapy, personal or logistic reasons. Some studies showed that a prolonged interval between neoadjuvant therapy and esophagectomy resulted in similar outcomes (Kim et al. 2012; Kathiravetpillai et al. 2016; Nilsson et al. 2020). Several studies reported that a prolonged interval between neoadjuvant therapy and esophagectomy was associated with a higher pathological response rate and similar long-term survival (Shapiro et al. 2014; Lee et al. 2016; Klevebro et al. 2020). Several studies revealed increased pathological response with prolonged interval, but worse long-term survival (Levinsky et al. 2020; Franko et al. 2016; Ranney et al. 2017). Additionally, some studies found that a prolonged interval following neoadjuvant therapy before esophagectomy was associated with increased incidence of postoperative complications (Teman et al. 2013), increased mortality (Wang et al. 2015), and poorer long-term survival (Chidambaram et al. 2023). Therefore, we launched this retrospective study to explore whether time interval from neoadjuvant therapy to surgery affect outcomes for locally advanced ESCC. And the cut off of 8 weeks were usually reserved to distinguish between early surgery group and delayed surgery group (Tie et al. 2018; Qin et al. 2018; Shang et al. 2020; Karthyarth et al. 2023). Therefore, we set the interval time at 8 weeks in the current study.

## Methods

### Study design and patients

Our study was a retrospective study, which consecutively enrolled ESCC patients who received esophagectomy after neoadjuvant camrelizumab combined with chemotherapy at the Department of Thoracic Surgery, the First Affiliated Hospital, Zhejiang University School of Medicine. It had been permitted by the Clinical Research Ethics Committee of the First Affiliated Hospital of Zhejiang University School of Medicine (2021 IIT No. 742) and was in line with the Helsinki Declaration (revised in 2013) and Good Clinical Practice Guidelines.

Inclusion criteria included histopathologically diagnosed ESCC by gastroscopy, pre-treatment clinical stage II-IVA (according to the eighth edition of the AJCC TNM staging (Rice et al. 2017)), receipt of 2–4 cycles (3 weeks per cycle) of neoadjuvant camrelizumab (200 mg) combined with platinum-containing dualdrug chemotherapy (platinum + paclitaxel), age over 18 and under 80 years and Eastern Cooperative Oncology Group (ECOG) performance status of 0 or 1. Exclusion criteria were incomplete information at our hospital, previous anticancer treatment (such as radiotherapy, interventional therapy or drug treatment), autoimmune disease or infectious disease, ongoing systemic immunosuppressive treatment, other malignant tumors and distant metastases. These patients were split up into two groups according to time interval from neoadjuvant immunochemotherapy to surgery: ≤ 8 weeks group (*n* = 44) and > 8 weeks group (*n* = 36).

### Treatment procedures and data collection

Immunotherapy regimen was camrelizumab 200 mg. Chemotherapy regimen included platinum (75 mg/m^2^ of cisplatin, or area under the curve (AUC) of the plasma concentration–time curve after a single dose = 5 of carboplatin, or 80 mg/m^2^ of nedaplatin) and paclitaxel (260 mg/m^2^ of albumin-bound paclitaxel). Before neoadjuvant treatment, systematic imaging evaluations were performed for all patients, including computed tomography (CT) of the esophagus, endoscopic ultrasound, positron emission tomography (PET)–CT, brain magnetic resonance imaging and abdominal ultrasound. During neoadjuvant therapy, CT of the esophagus was performed every 2 cycles until the patient underwent surgery or withdrew from treatment. Moreover, routine blood and biochemical blood examinations were conducted every week. And myocardial enzyme spectrum, thyroid function, and coagulation function examinations were done every 3 weeks. We evaluated patients’ gastrointestinal reactions and skin reactions by their complaints. The response evaluation criteria in solid tumor version 1.1 (RECIST 1.1) (Eisenhauer et al. 2009) was used to evaluate the tumor treatment response–complete response (CR): disappearance of all target lesions, partial remission (PR): ≥ 30% decline in the total diameter of target lesions, progressive disease (PD): ≥ 20% enlargement in the total diameter of target lesions or the appearance of new lesions, stable disease (SD): neither CR, PR nor PD. Objective response rate (ORR) included CR and PR. Adverse events (AEs) were graded on the basis of Common Terminology Criteria for Adverse Events (CTCAE) version 5.0 (Common Terminology Criteria for Adverse Events (CTCAE), 2017).

Surgical approaches included open radical surgery, video-assisted thoracoscopic surgery (VATS), and robot-assisted thoracoscopic surgery (RATS). Surgical methods were comprised of Mc-Kewon and Ivor-Lewis. We considered Ivor-Lewis esophagectomy with at least a two-field lymph node dissection for inferior and medial ESCC, and McKeown esophagectomy with three-field lymph node dissection (neck, thoracic and abdominal lymph nodes) for superior ESCC. We adopted tumor regression grade (TRG) to express pathological response. TRG was divided into four categories according to the College of American Pathologists (CAP)/The National Comprehensive Cancer Network (NCCN) guidelines: TRG 0 (no remaining active tumor cells), TRG 1 (residual viable tumor cells ≤ 10%), TRG 2 (10% < residual viable tumor cells ≤ 50%) and TRG 3 (remaining active tumor cells > 50%). The pathological complete remission (pCR) rate and major pathological response (MPR) rate were considered as equal to TRG 0 and TRG 0–1 respectively. Postoperative complications were evaluated based on definitions proposed by the Esophagectomy Complications Consensus Group (ECCG) (Low et al. 2015).

After surgery, imaging assessments were conducted every 1–3 months. Patients continued to receive chemotherapy plus camrelizumab after surgery until the full 6 cycles, and then continued to receive camrelizumab alone for 1–2 years or until disease progression. And the follow-up date would not end until at least 1 year after surgery. The primary endpoints of this study were DFS and OS. DFS was defined as the time from surgery to disease progression according to the RECIST 1.1 or death, whichever occurred first. OS was defined as the time from surgery until death from any cause. Secondary endpoints of this study were pathological response (MPR and pCR), surgical outcomes and postoperative complications.

### Statistical analysis

Categorical variables were expressed as frequencies (percentages), and continuous variables were shown as the median and interquartile range (IQR). Categorical variables were analyzed using the Chi-square test or Fisher’s exact test and continuous variables were compared with the t-test or Wilcoxon test. DFS and OS were estimated using the Kaplan–Meier method and compared with the stratified log-rank test. Median follow-up time was evaluated with the reverse Kaplan–Meier method. Stratified Cox proportional-hazards models were used to assess the correlation between each study variable and survival outcomes. Statistical analyses were performed using R software (version 4.1.2) and plotting was performed using GraphPad Prism version 9.0 (GraphPad Software, San Diego, CA, USA). A two-sided *P* value < 0.05 was considered to be statistically significant.

## Results

### Baseline characteristics

**Fig. 1:**
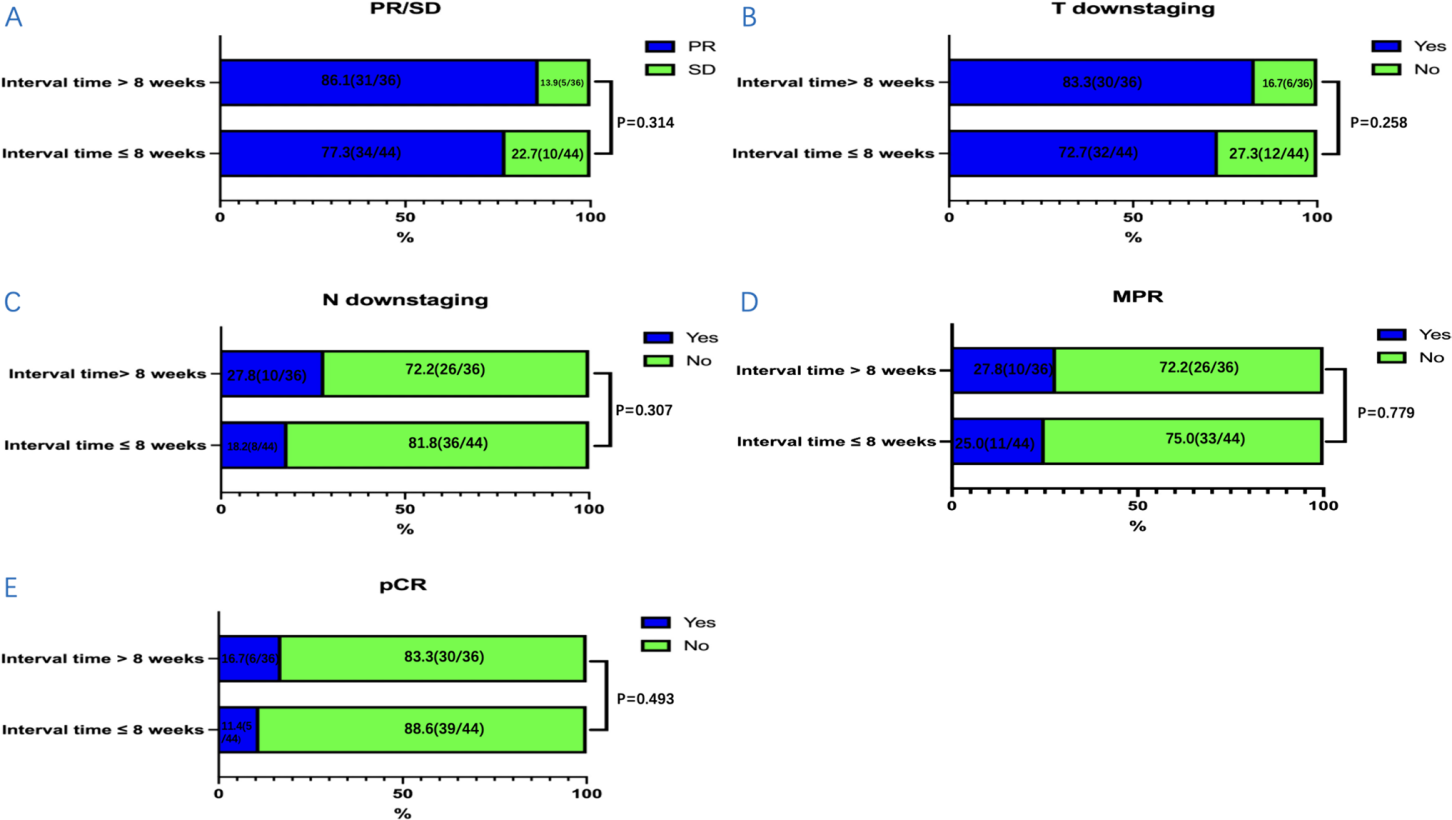
The distribution condition of clinical response, T downstaging, N downstaging and pathological response between the two groups: **A** PR/SD/PD, **B** T downstaging, **C** N downstaging, **D** MPR, and **E** pCR. Clinical response included partial remission (PR) and stable disease (SD). Pathological response included major pathological response (MPR) and pathological complete remission (pCR). T downstaging and N downstaging were assessed by CT before and after neoadjuvant immunochemotherapy. *CT* computed tomography

From 2019 to 2021, a total of 80 patients were included in our study and were divided into two groups according to the time interval from neoadjuvant immunochemotherapy to surgery: ≤ 8 weeks group (*n* = 44) and > 8 weeks group (*n* = 36). The median time to surgery was 51.0 days (IQR, 49.0–54.0 days) in the ≤ 8 weeks group and 96.0 days (IQR, 81.3–101.8 days) in the > 8 weeks group. Characteristics of these patients at baseline are shown in Table 1. There were no significant differences between the two groups in age, gender, ECOG performance status, smoking status, drinking status, comorbidities, pathological grade, tumor location, clinical stage, and treatment cycle. The ORR in the ≤ 8 weeks group was 77.3% and 86.1% in the > 8 weeks group (*P* = 0.314, Fig. 1A). The rate of T downstaging (assessed by CT before and after neoadjuvant immunochemotherapy) was 72.7% and 83.3% in the ≤ 8 weeks group and the > 8 weeks group, respectively (*P* = 0.258, Fig. 1B). The rate of N downstaging (assessed by CT before and after neoadjuvant immunochemotherapy) in the ≤ 8 weeks group was 18.2% and 27.8% in the > 8 weeks group (*P* = 0.307, Fig. 1C).

**Table 1:** Characteristics of the patients at baseline

Variables	Total, *n* = 80	≤ 8 weeks, *n* = 44	> 8 weeks, *n* = 36	*P* value
Age (years), median age (IQR)	66.0 (57.3–70.0)	65.0 (56.0–70.0)	67.5 (59.0–71.8)	0.377
Gender, *n* (%)				0.500
Male	72 (90.0)	41 (93.2)	31 (86.1)	
Female	8 (10.0)	3 (6.8)	5 (13.9)	
ECOG performance status, *n* (%)				0.428
0	45 (56.3)	23 (52.3)	22 (61.1)	
1	35 (43.7)	21 (47.7)	14 (38.9)	
Smoking status, *n* (%)				0.840
Never	39 (48.8)	21 (47.7)	18 (50.0)	
Ever	41 (51.2)	23 (52.3)	18 (50.0)	
Drinking status, *n* (%)				0.369
Never	40 (50.0)	20 (45.5)	20 (55.6)	
Ever	40 (50.0)	24 (54.5)	16 (44.4)	
Comorbidities, *n* (%)				
Diabetes mellitus	4 (5.0)	3 (6.8)	1 (2.8)	0.757
Hypertension	20 (25.0)	11 (25.0)	9 (25.0)	1.000
Pathological grade, *n* (%)				0.519
G1	3 (3.8)	2 (4.5)	1 (2.8)	
G2	43 (53.8)	26 (59.1)	17 (47.2)	
G3	22 (27.5)	9 (20.5)	13 (36.1)	
Unknown	12 (15.0)	7 (15.9)	5 (13.9)	
Tumor location, *n* (%)				0.408
Locus superior	11 (13.8)	5 (11.4)	6 (16.7)	
Locus medialis	43 (53.8)	22 (50.0)	21 (58.3)	
Locus inferior	26 (32.5)	17 (38.6)	9 (25.0)	
cT stage, *n* (%)				0.446
T2	9 (11.3)	6 (13.6)	3 (8.3)	
T3	44 (55.0)	21 (47.7)	23 (63.9)	
T4a	6 (7.5)	3 (6.8)	3 (8.3)	
T4b	21 (26.3)	14 (31.8)	7 (19.4)	
cN stage, *n* (%)				0.654
N0	10 (12.5)	7 (15.9)	3 (8.3)	
N1	25 (31.3)	14 (31.8)	11 (30.6)	
N2	42 (52.5)	22 (50.0)	20 (55.6)	
N3	3 (3.8)	1 (2.3)	2 (5.6)	
cStage, *n* (%)				0.621
II	9 (11.3)	6 (13.6)	3 (8.3)	
III	42 (52.5)	21 (47.7)	21 (58.3)	
IVA	29 (36.3)	17 (38.6)	12 (33.3)	
Treatment cycle, *n* (%)				0.459
2	20 (25.0)	11 (25.0)	9 (25.0)	
3	32 (40.0)	20 (45.5)	12 (33.3)	
4	28 (35.0)	13 (29.5)	15 (41.7)	

*IQR* interquartile range, *ECOG* Eastern Cooperative Oncology Group, *PS* performance status

### Adverse events

There were no previously unrecorded AEs in our study. Grade 3–4 AEs of neoadjuvant therapy were summarized in Table 2. The incidence of grade 3–4 AEs in the ≤ 8 weeks group was 27.3% and 36.1% in the > 8 weeks group. Grade 3–4 AEs were mainly distributed in hematological abnormalities (anemia). There were no significant differences in the occurrence of grade 3–4 AEs between the two groups. These AEs were quickly resolved after symptomatic treatment.

**Table 2:** Grade 3–4 AEs of neoadjuvant immunochemotherapy

Event	Total, *n* = 80	≤ 8 weeks, *n* = 44	> 8 weeks, *n* = 36	*P* value
Any AEs	25 (31.3)	12 (27.3)	13 (36.1)	0.396
Hematologic				
Leukopenia	5 (6.3)	2 (4.5)	3 (8.3)	0.816
Agranulocytosis	4 (5.0)	1 (2.3)	3 (8.3)	0.470
Anemia	16 (20.0)	6 (13.6)	10 (27.8)	0.116
Thrombocytopenia	2 (2.5)	0 (0.0)	0 (0.0)	NA
Gastrointestinal				
Nausea	0 (0.0)	0 (0.0)	0 (0.0)	NA
Emesis	0 (0.0)	0 (0.0)	0 (0.0)	NA
Diarrhea	0 (0.0)	0 (0.0)	0 (0.0)	NA
Constipation	0 (0.0)	0 (0.0)	0 (0.0)	NA
Hepatic injury	4 (5.0)	3 (6.8)	1 (2.8)	0.757
Renal injury	0 (0.0)	0 (0.0)	0 (0.0)	NA
Skin reaction	3 (3.8)	0 (0.0)	3 (8.3)	0.174
Hypothyroidism	0 (0.0)	0 (0.0)	0 (0.0)	NA
Coagulation disorders	0 (0.0)	0 (0.0)	0 (0.0)	NA
Esophageal fistula	0 (0.0)	0 (0.0)	0 (0.0)	NA

*AEs* adverse events

### Surgical outcomes and pathological response

The outcomes of surgery and the pathological response were summarized in Table 3. There were no significant differences in the surgical approach, operation time, blood loss, length of hospital stays, TRG, and ypTNM stage between the two groups. The rate of R0 resection was 100.0% in the ≤ 8 weeks group and 97.2% in the > 8 weeks group. And more lymph nodes were removed during surgery in the ≤ 8 weeks group compared with the > 8 weeks group (*P* = 0.034). The rate of MPR in the ≤ 8 weeks group was 25.0% and 27.8% in the > 8 weeks group (*P* = 0.779, Fig. 1D). The rate of pCR in the ≤ 8 weeks group was 11.4%, with 16.7% in the > 8 weeks group (*P* = 0.493, Fig. 1E). Overall, the incidence of postoperative complications in the ≤ 8 weeks group was 27.3% and 19.4% in the > 8 weeks group (*P* = 0.413). There were no significant differences in the postoperative complications and no perioperative deaths occurred.

**Table 3:** Surgical outcomes and pathological response

Variables	Total, *n* = 80	≤ 8 weeks, *n* = 44	> 8 weeks, *n* = 36	*P* value
Surgical approach, *n* (%)				0.461
Open	30 (37.5)	19 (43.2)	11 (30.6)	
VATS	44 (55.0)	21 (47.7)	23 (63.9)	
RATS	5 (6.3)	3 (6.8)	2 (5.6)	
VATS-open	1 (1.3)	1 (2.3)	0 (0.0)	
Operation time, median (IQR), min	280.0 (248.5–319.0)	274.0 (231.0–324.0)	288.0 (263.0–318.0)	0.265
Estimated blood loss, median (IQR), mL	100.0 (50.0–100.0)	100.0 (50.0–100.0)	100.0 (50.0–100.0)	0.649
Resection margin, *n* (%)				0.919
R0	79 (98.8)	44 (100.0)	35 (97.2)	
R1	1 (1.3)	0 (0.0)	1 (2.8)	
Number of lymph node dissections during surgery, median (IQR), *n* (%)	21.0 (12.0–30.0)	21.0 (12.0–30.0)	19.0 (13.0–28.0)	0.034
Length of hospital stay, median (IQR), day	19.0 (14.0–23.0)	20.0 (15.0–23.0)	16.0 (13.0–22.0)	0.618
Postoperative complication, *n* (%)				
Overall	19 (23.8)	12 (27.3)	7 (19.4)	0.413
Aspiration pneumonia	6 (7.5)	2 (4.5)	4 (11.1)	0.495
Anastomotic leak	4 (5.0)	2 (4.5)	2 (5.6)	1.000
Tracheoesophageal fistula	1 (1.3)	0 (0.0)	1 (2.8)	1.000
Chyle leak	1 (1.3)	1 (2.3)	0 (0.0)	1.000
Anastomotic stenosis	1 (1.3)	1 (2.3)	0 (0.0)	1.000
Gastroparesis	1 (1.3)	1 (2.3)	0 (0.0)	1.000
Intestinal obstruction	2 (2.5)	1 (2.3)	1 (2.8)	1.000
Diaphragmatic paralysis	1 (1.3)	1 (2.3)	0 (0.0)	1.000
Delayed incision healing	1 (1.3)	1 (2.3)	0 (0.0)	1.000
Postoperative bleeding	1 (1.3)	1 (2.3)	0 (0.0)	1.000
Pathological grade, *n* (%)				0.605
TRG0	11 (13.8)	5 (11.4)	6 (16.7)	
TRG1	10 (12.5)	6 (13.6)	4 (11.1)	
TRG2	43 (53.8)	26 (59.1)	17 (47.2)	
TRG3	16 (20.0)	7 (15.9)	9 (25.0)	
ypTNM stage				0.661
0	11 (13.8)	5 (11.4)	6 (16.7)	
I	6 (7.5)	3 (6.8)	3 (8.3)	
II	22 (27.5)	15 (34.1)	7 (19.4)	
IIIA	7 (8.8)	3 (6.8)	4 (11.1)	
IIIB	33 (41.3)	17 (38.6)	16 (44.4)	
IVA	1 (1.3)	1 (2.3)	0 (0.0)	

*IQR* interquartile range, *VATS* video-assisted thoracoscopic surgery, *RATS* robot-assisted thoracoscopic surgery, *TRG* tumor regression grade

### Survival

At the time of data cutoff (December 2023), the median follow-up time for the ≤ 8 weeks group was 35.7 months (95% confidence interval [CI] 32.5–39.0), while the median follow-up time for the > 8 weeks group was 31.0 months (95% CI 24.8–37.3). Among the ≤ 8 weeks group, 18.2% (8/44) patients experienced recurrence and metastasis, and 7 patients died due to recurrence and metastasis. Among the > 8 weeks group, 38.9% (14/36) patients experienced recurrence and metastasis, 1 patient died from COVID-19, and 14 patients died due to cancer recurrence and metastasis. The summary of recurrence and metastasis in the two groups is shown in Table 4.

**Table 4:** Disease recurrence and metastasis

Recurrence and metastasis	≤ 8 weeks, *n* = 44	> 8 weeks, *n* = 36	*P* value
Overall	8 (18.2)	14 (38.9)	0.039
Locoregional recurrence	1 (2.3)	3 (8.3)	0.470
Metastasis distant			
Brain	1 (2.3)	3 (8.3)	0.470
Liver	0 (0.0)	2 (5.6)	0.388
Bone	4 (7.0)	2 (5.6)	0.865
Lung	0 (0.0)	3 (8.3)	0.174
Lymph node	1 (2.3)	0 (0.0)	1.000
Renicapsule	1 (2.3)	1 (2.8)	1.000

**Fig. 2:**
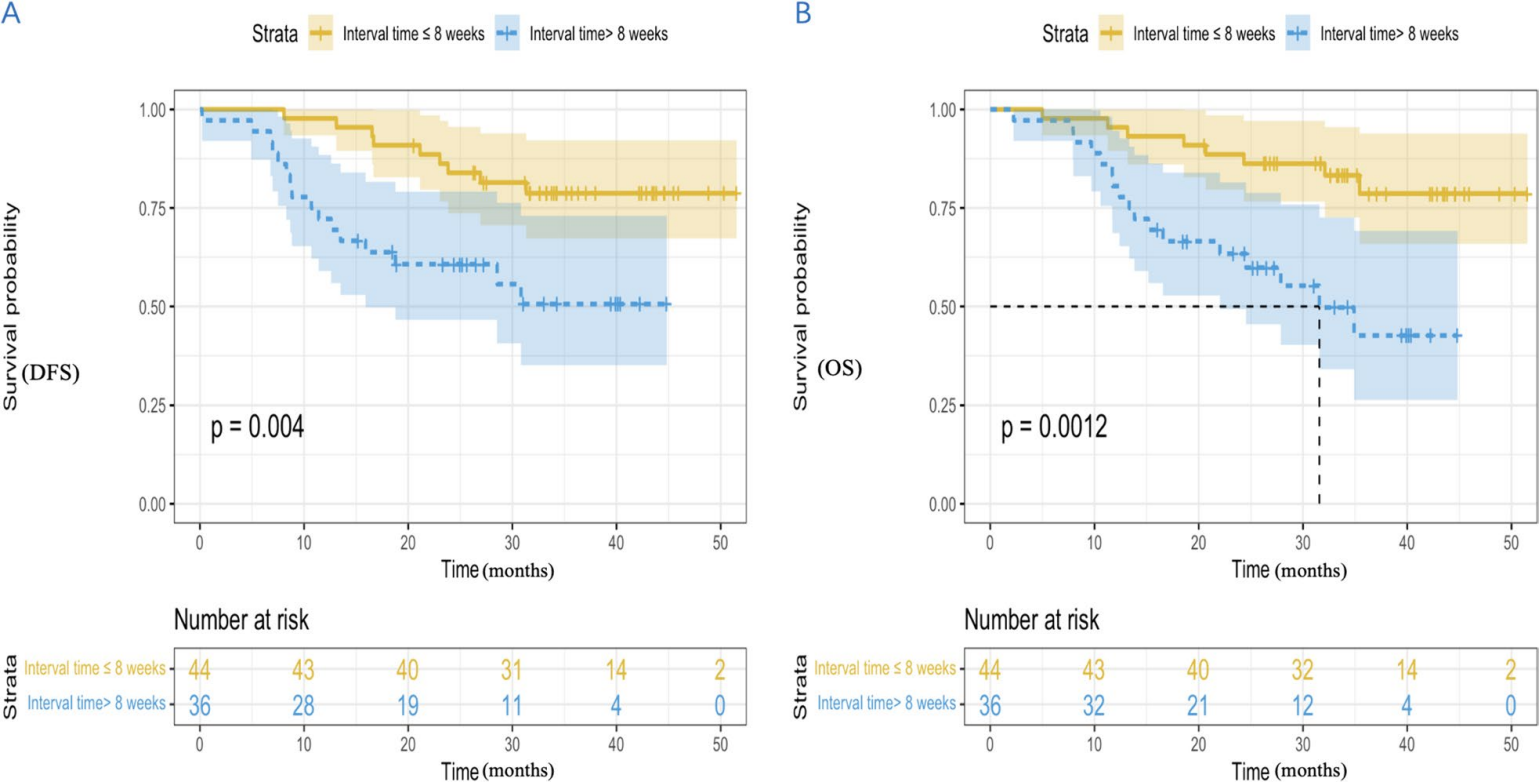
Kaplan Meier curves of DFS (**A**) and OS (**B**) between the two groups. DFS, disease-free survival; OS, overall survival

The median DFS in the two groups had not yet reached (hazard ratio [HR], 3.153; 95% CI 1.383 to 6.851; *P* = 0.004) (Fig. 2A). The 1-year DFS rate, 2-year DFS rate, and 3-year DFS rate in the ≤ 8 weeks group were 97.7%, 84.1%, and 79.5%, with that in the > 8 weeks group 72.2%, 61.1% and 55.6%. The median OS of ≤ 8 weeks group was not achieved (HR, 3.703; 95% CI 1.584 to 8.657; *P* = 0.0012), with the > 8 weeks group 31.6 months (95% CI 21.1 to 42.1) (Fig. 2B). The 1-year OS rate, 2-year OS rate and 3-year OS rate in the ≤ 8 weeks group were 95.5%, 88.6% and 81.8%, with that in the > 8 weeks group 80.6%, 63.9% and 52.8%.

**Fig. 3:**
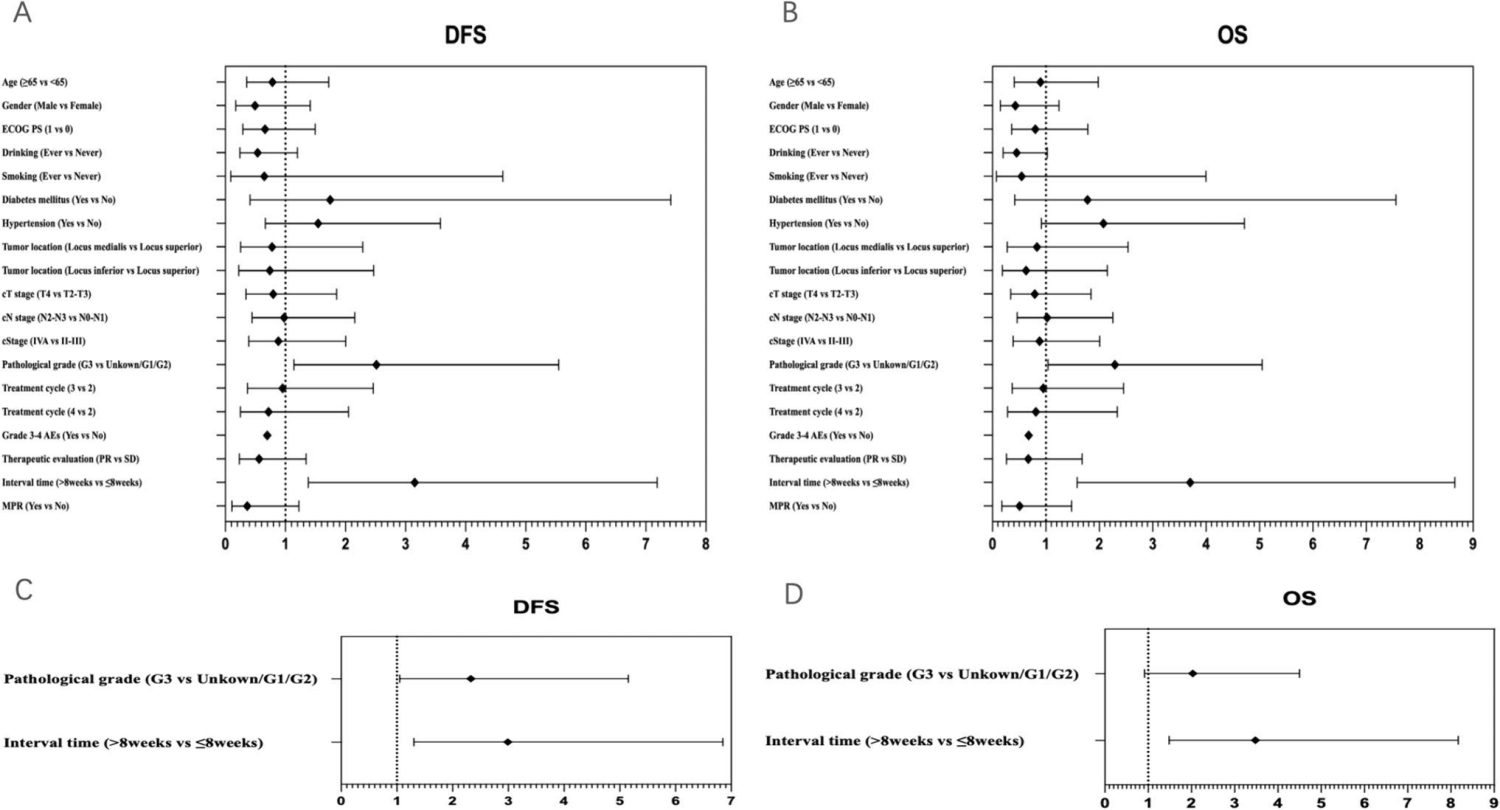
Forest plot of hazard ratio of univariable and multivariable Cox regression analyses for DFS (**A**, **C**) and OS (**B**, **D**). *DFS*, disease-free survival, *OS* overall survival, *ECOG PS* eastern cooperative oncology group performance status, *PR* partial remission, *SD* stable disease, *AEs* adverse events, *MPR* major pathologic response

In univariable Cox regression analyses, there were no statistically significant correlations between these included factors and DFS (Fig. 3A) or OS (Fig. 3B), except for pathological grade and interval time. Patients with G3 had inferior DFS and OS (HR, 2.516; 95% CI 1.141 to 5.548; and HR, 2.292; 95% CI 1.040 to 5.051, respectively). Interval time > 8 weeks was associated with inferior DFS and OS (HR, 3.153; 95% CI 1.383 to 6.851; and HR, 3.703; 95% CI 1.584 to 8.657, respectively). Moreover, we performed multivariable Cox regression analyses on statistically significant factors identified through univariable analyses (Fig. 3C and D). Inferior DFS and OS were observed in patients with interval time > 8 weeks (HR, 2.992; 95% CI 1.306 to 6.851; and HR, 3.478; 95% CI 1.481 to 8.170, respectively). Patients with G3 were associated with inferior DFS (HR, 2.327; 95% CI 1.051 to 5.152), but not inferior OS (HR, 2.032; 95% CI 0.919 to 4.496). It can be seen that interval time ≤ 8 weeks independently predicted better survival.

## Discussion

Nowadays, there is still controversy over the outcomes of prolonged time intervals from neoadjuvant therapy to surgery for locally advanced esophageal squamous cell carcinoma (ESCC) (Kathiravetpillai et al. 2016; Shapiro et al. 2014; Franko et al. 2016; Teman et al. 2013). Therefore, we launched this retrospective study to explore whether the time interval from neoadjuvant therapy to surgery affects outcomes for locally advanced ESCC. Moreover, there is currently no consensus on the optimal interval time between neoadjuvant therapy and surgery. In clinical practice, the interval time has usually been set at 4 to 8 weeks (Yang et al. 2018; Haisley et al. 2016). In the current study, we set the interval time at 8 weeks. We found that the time interval from neoadjuvant camrelizumab combined with chemotherapy to surgery > 8 weeks was not associated with a difference in postoperative complications, postoperative morbidity, and pathological response. However, delaying surgery increases the risk of recurrence and metastasis for locally advanced ESCC patients. A longer interval between neoadjuvant therapy and surgery (> 8 weeks) was associated with worse long-term survival. Despite no significant differences in clinical oncologic factors (cStage) or surgical outcomes (R0 rate, complication) and tumor evaluation variables (pCR, TRG) between the two groups, the prognosis was poor in the surgery group after 8 weeks. In my opinion, the reasons for this result are as listed. Firstly, a longer waiting period may increase the risk of tumor repopulation, recurrence, and metastasis (Tessier et al. 2014; Chiu et al. 2013). Secondly, a longer waiting period was not a result of the patient’s preferences or opportunities, but rather because of their poor physical condition after neoadjuvant therapy, which may result in an inherent disadvantage in terms of survival. Finally, apart from clinical oncologic factors (cStage) or surgical outcomes (R0 rate, complication) and tumor evaluation variables (pCR, TRG), different factors have a significant impact on OS. Due to the various confounding factors of this issue, it may be necessary to conduct prospective randomized studies.

The findings of our study were different from the findings of other studies. Two studies and a meta-analysis showed there was no significant difference in the pathologic response and overall survival between timely esophagectomy and delayed esophagectomy (Kim et al. 2012; Tie et al. 2018; Tessier et al. 2014). A meta-analysis revealed a longer interval associated with unchanged pathological response and reduced overall survival (Lin et al. 2016). Three studies found a prolonged interval was associated with higher pathological response, without affecting survival (Haisley et al. 2016; Shapiro et al. 2014; Lee et al. 2016). Levinsky et al. and a meta-analysis showed that the delayed esophagectomy group (interval ≥ 90 days) had higher rates of pathological complete response and poorer overall survival (Levinsky et al. 2020; Qin et al. 2018). In our study, we found that there was no significant difference in the pathological response. The rates of MPR and pCR in the ≤ 8 weeks group and > 8 weeks group were similar (25.0% vs 27.8%, 11.4% vs 16.7%, *P* > 0.05). A longer interval (> 8 weeks) was associated with worse long-term survival. The median DFS in the two groups had not yet reached (*P* = 0.004). The median OS of the ≤ 8 weeks group was not achieved (*P* = 0.0012), with the > 8 weeks group at 31.6 months. The reasons for these differences may be different interval time, different neoadjuvant therapy regimens, and different treatment cycles.

In the study, we found that pathological grade (G3) and interval time > 8 weeks were associated with inferior DFS and OS in univariable Cox regression analyses. And after multivariable Cox regression analyses, inferior DFS and OS were observed in patients with interval time > 8 weeks. It can be seen that interval time ≤ 8 weeks independently predicted better survival. Therefore, it is not reasonable to delay esophagectomy beyond 8 weeks for patients who can tolerate surgery. However, patients with G3 were associated with inferior DFS (HR, 2.327; 95% CI 1.051 to 5.152), but not inferior OS (HR, 2.032; 95% CI 0.919 to 4.496). The reason for this may be the small sample size. Larger samples and randomized controlled trials are needed to confirm. Additionally, we found more lymph nodes were removed during surgery in the ≤ 8 weeks group compared with the > 8 weeks group (*P* = 0.034). The reason we speculated was that delaying surgery made surgical dissection more difficult. A longer waiting period may lead to tumor repopulation or increase fibrosis and adhesion. In our study, there were no significant differences in the postoperative complications and no perioperative deaths occurred. The incidence of postoperative complications varied in different clinical researches. Nilsson et al. and Tie et al. found there were no significant differences in postoperative complications and 90-day mortality (Nilsson et al. 2020; Tie et al. 2018). Chidambaram et al. and Karthyarth et al. revealed that delay in surgery was associated with higher mortality and complications rates (Chidambaram et al. 2023; Karthyarth et al. 2023).

### There are some limitations in this study

Firstly, our study is a retrospective study. The patients may be allocated to the two groups in a non-randomized manner. This may result in potential bias. And our sample size was small. This may limit our statistical ability for research. Therefore, our findings require larger scale randomized controlled trials to validate. Secondly, there was heterogeneity in patients in our study and our findings were based on a post-hoc analysis, which may cause some impacts on the results. Moreover, delaying surgery after neoadjuvant therapy is inevitable owing to adverse events of neoadjuvant therapy, poor physical condition, personal or logistic reasons. This may result in impacts in terms of survival. And the cutoff point of the interval was different in different studies. In the current study, we set the interval time at 8 weeks. This may result in potential bias. Finally, the postoperative follow-up time of this study was relatively short. Therefore, further follow-up actions are needed to evaluate long-term outcomes.

In conclusion, prolonged time interval from neoadjuvant camrelizumab combined with chemotherapy to surgery may increase the risk of recurrence and metastasis for locally advanced ESCC patients. And a longer interval time (> 8 weeks) was associated with worse long-term survival, but similar pathological response rate. It is not reasonable to delay esophagectomy beyond 8 weeks for patients who can tolerate surgery.

## Data Availability

The data of the current study are available from the corresponding author on reasonable request.
